# MultiScale-CNN-4mCPred: a multi-scale CNN and adaptive embedding-based method for mouse genome DNA N4-methylcytosine prediction

**DOI:** 10.1186/s12859-023-05135-0

**Published:** 2023-01-18

**Authors:** Peijie Zheng, Guiyang Zhang, Yuewu Liu, Guohua Huang

**Affiliations:** 1 School of Information Engineering, Shaoyang University, Shaoyang, 42200 China; 2 College of Information and Intelligence, Hunan Agricultural University, Changsha, 410128 China

**Keywords:** Deep learning, Convolutional neural network, Long short-term memory, Embedding, N4-Methylcytosine

## Abstract

N4-methylcytosine (4mC) is an important epigenetic mechanism, which regulates many cellular processes such as cell differentiation and gene expression. The knowledge about the 4mC sites is a key foundation to exploring its roles. Due to the limitation of techniques, precise detection of 4mC is still a challenging task. In this paper, we presented a multi-scale convolution neural network (CNN) and adaptive embedding-based computational method for predicting 4mC sites in mouse genome, which was referred to as MultiScale-CNN-4mCPred. The MultiScale-CNN-4mCPred used adaptive embedding to encode nucleotides, and then utilized multi-scale CNNs as well as long short-term memory to extract more in-depth local properties and contextual semantics in the sequences. The MultiScale-CNN-4mCPred is an end-to-end learning method, which requires no sophisticated feature design. The MultiScale-CNN-4mCPred reached an accuracy of 81.66% in the 10-fold cross-validation, and an accuracy of 84.69% in the independent test, outperforming state-of-the-art methods. We implemented the proposed method into a user-friendly web application which is freely available at: http://www.biolscience.cn/MultiScale-CNN-4mCPred/.

## Background

DNA modifications play a crucial role in some biological processes and diseases, including development [1], aging [2], cancer [3], and regulation of gene expression [4]. Over 17 types of chemical modifications have been identified in DNA so far [5], including 5-methylcytosine (5mC), 5-hydroxymethylcytosine (5hmC), 5-formylcytosine (5fC), 5-carboxylcytosine (5caC), and N6-methyladenine (6mA) [6, 7]. DNA methylation is an important epigenetic modification, which is catalyzed by a family of DNA methyltransferases. DNA methylation is involved in heavy metal modification and regulation of chromatin as well as gene expression [8]. Methylation has widely been found in prokaryotic and eukaryotic genomes, including N4-methylcytosine (4mC) [9], 5-methylcytosine (5mC) [10], and N6-methyladenine (6mA) [11, 12]. The 5mC is formed by transferring a methyl group from S-adenyl methionine to the fifth carbon of a cytosine residue [13] and has been widely demonstrated to play an important role in the biological progression associated with diabetes, cancer, and some neurological diseases [14–16]. The well-known DNA 6mA is a process of adding a methyl group to the 6-th position of an adenine ring catalyzed by DNA methyltransferases [17]. The 6mA is an essential epigenetic modification. The 4mC formation is catalyzed by N4-cytosine-specific DNA methyltransferases, which methylate the amino group at the fourth position of cytosine [18]. The 4mC is involved in such biological functions as host defense, transcription regulation, gene expression, and DNA replication [19]. In comparison with the 5mC and the 6mA, little was known about the 4mC modification and functions.

Detecting 4mC sites is critical to exploring 4mC functions. Many experimental methods have been developed to detect 4mC sites over the past decade, including single-molecule real-time sequencing (SMRT) and bisulfite sequencing [20]. These experimental methods generally are labor-intensive and time-consuming. The emergence of bioinformatics makes it possible to identify 4mC sites on a large scale. Hundreds of computational tools have been developed over the past thirty years for predicting post-translational modification [17, 21–23], RNA modification [24–26], single-cell analysis [27], protein functions [28, 29], as well as protein structure [30], gene selection [31], cancer diagnosis [32], and even food recommendation [33]. With the help of computers of powerful computational ability, the computational methods made great progress in the protein structure recognition, which is thought as one of best challenging tasks. AlphaFold [30] is approaching accuracy of the experimentally determining protein structure. The vast achievement attributed to design of machine learning algorithm, which refined high level representation of protein structure.

Following these successes, many computational tools have been developed for predicting 4mC sites [21, 26, 34, 35]. iDNA4mC [9], Meta-4mCpred [34], and 4mCPred [26] were designed to predict 4mC sites for six species: Arabidopsis thaliana (A. thaliana), Caenorhabditis elegans (C. elegans), Drosophila melanogaster (D. melanogaster), Escherichia coli (E. coli), Geobacter pickeringii (G. pickeringii) and Geoalkalibacter subterraneus (G. subterraneus). These methods are simple to use, but their capacity of detecting 4mC sites cross species need promoting. No less than five computational methods [36–41] were intended to predict 4mC sites in mouse genomes. Both 4mCPred-EL [36] and i4mC-Mouse [37] were feature engineering-based methods. 4mCPred-EL [36] integrated probabilities outputted by 28 classifiers which were generated by combining four common machine learning algorithms with seven types of features. The i4mC-Mouse [37] also used multiple representations and selected informative representations for detecting 4mC sites. The 4mCPred-CNN [38] and the Mouse4mC-BGRU [39] were deep learning-based schemes, which were quite different from the two methods above. The 4mCPred-CNN [38] used one-hot encoding and convolutional neural networks (CNNs), while the Mouse4mC-BGRU [39] utilized the adaptive embedding and bidirectional gated recurrent units (GRU). The deep learning-based methods exhibited a superiority over the 4mCPred-EL [36] and the i4mC-Mouse [37] in the empirical experiments. In addition, the 4mCPred-CNN [38] and the Mouse4mC-BGRU [39] were an end-to-end learning method that takes primary sequences as input, and outputs directly predictive results, requiring no feature extraction.

Although vast efforts were paid to promote the predictive accuracy of identifying 4mC sites, there are some drawbacks to overcome. Representations of sequence are critical in the feature engineering-based methods. Most representations are degenerative mapping. That’s to say, during the course of converting sequences into representation, there would be information lost. Ensemble of more representations would make the model improve predictive accuracy, but added the computational costs. Most representations are based on character statistics, disregarding the relationship between characters, namely contextual semantics. The deep learning-based methods are very promising to promote significantly predictive accuracy of 4mC recognitions. CNNs are good at characterizing local property, while the GRU and the long short-term memory (LSTM) do well in capturing contextual semantics. Different scale CNN reflects different scale information of sequences. The 4mCPred-CNN [38] failed to exploit multi-scale information.

To overcome the drawbacks above we proposed a deep neural network-based computational method named MultiScale-CNN-4mCPred for mouse 4mC site prediction. The MultiScale-CNN-4mCPred consisted of adaptive embedding, bidirectional LSTM (Bi-LSTM) followed by three scale CNNs, and fully-connected network. DNA sequences resemble sentences in the natural language, and thus there would be semantics hidden in it. The LSTM was intended to extract the semantics of 4mC DNA sequences, and the multi-scale CNN was intended to extract sequence representation at a different scale. The highlight of the MultiScale-CNN-4mCPred was summarized as follows. Firstly, the MultiScale-CNN-4mCPred combined the multi-scale CNNs and Bi-LSTM, and used them to capture scale representations and semantics respectively, which promoted predictive accuracy. The 4mCPred-CNN [38] didn’t use LSTM or GRU to capture semantics, while the Mouse4mC-BGRU [39] didn’t exploit CNNs to characterize local property. Secondly, the MultiScale-CNN-4mCPred used the adaptive embedding which is dynamic to represent sequence with comparison to one-hot encoding.

## Results

### Optimizing combination of different scale CNNs

Multiscale refers to signal sampling at different scales, and different characteristics can be observed at different scales. Larger scale characterizes global information, while the smaller scale reflects local characteristics. Therefore, the CNNs with different scales are of capacity to capture different information. We investigated the effect of the combination of different scale CNNs on the performances. We randomly selected 80% samples of the training dataset as the training samples and the remaining 20% as the validation set. Table 1 showed the performances of the combination of different scale CNNs. It was obviously observed that the combination of CNNs with the kernel sizes 3, 5, and 7 substantially outperformed others, indicating that the combination of these three scales can characterize well global, medium, and local structure. Therefore, we chose the above three scales for later experiments.

**Table 1:** The performance of different scale combinations

	Sn	Sp	Acc	MCC
1,3,5	0.7651	0.7867	0.7759	0.5519
**3,5,7**	**0.8188**	0.7867	**0.8027**	**0.6057**
5,7,9	0.7852	0.7800	0.7826	0.5652
7,9,11	0.7517	**0.8267**	0.7893	0.5801
11,13,15	0.7114	0.8333	0.7726	0.5490
13,15,17	0.7517	0.8200	0.7860	0.5731

The bold highlighted the best values in the column

### Comparison with different embedding

For text sequences, there are many frequently used embedding to represent it such as one-hot encoding and Word2Vec [42, 43]. One-hot encoding is well-known encoding in the natural language processing, where a word corresponds a 0/1 binary vector. One-hot encoding is unable to capture textual semantic relationship between words. Word2vec translated each word into a contiguous vector which contains intrinsic semantics in the sequence. We compared the adaptive embedding with two types of embedding. In the Word2Vec, we considered 1, 2, and 3 contiguous nucleotides as a word respectively. Except for the embedding, the other parts of model are identical. As shown in Table 2, the overall performance of the adaptive embedding over the validation set was the best, with three indices (Sn, Acc, and MCC) to be in the lead. This is a reason to choose the adaptive embedding, not One-hot or Word2Vec.

**Table 2:** Comparison between different embedding

	Sn	Sp	Acc	MCC
One-hot	0.7584	**0.8400**	0.7993	0.6005
Word2Vec(1)	0.7315	0.8133	0.7726	0.5468
Word2Vec(2)	0.8188	0.7600	0.7893	0.5797
Word2Vec(3)	0.7248	0.8200	0.7726	0.5474
Adaptive Embedding	**0.8456**	0.7800	**0.8127**	**0.6269**

The bold highlighted the best values in the column

## Discussion

### Comparison with state-of-the-art methods

As mentioned previously, many computational methods have been developed to predict DNA N4-methylcytosine in mouse genome over the recent ten years, such as Mouse4mC-BGRU [39], i4mC-Mouse [37], and 4mCPred-EL [36]. We performed the 10-fold cross-validation and the independent test to compare them. In the 10-fold cross-validation, the training set was randomly divided into ten parts in equal or approximate size. Nine parts were used for training the model, and the remaining one part for testing the trained model. This process was repeated ten times. In the independent test, the whole training set was used for training the model, and the testing set was used for testing the trained model. As shown in Table 3, Our MultiScale-CNN-4mCPred reached competitive performances with these state-of-the-art methods over the 10-fold cross-validation. For example, the MultiScale-CNN-4mCPred obtained the best Acc, the second-best MCC as well as Sn, and the third-best Sp. In addition, our method was more tradeoff between Sn and Sp than other methods, i.e., both Sn and Sp exceeded 0.8. Table 4 listed the performances over the independent test. Except for Sp, all indices of the MultiScale-CNN-4mCPred were best. The MultiScale-CNN-4mCPred increased Sn by 0.0563 over the Mouse4mC-BGRU [39], by 0.0492 over the i4mC-Mouse [37], and by 0.0991 over the 4mCpred-EL [36]. The MultiScale-CNN-4mCPred increased Acc by 0.0219 over the Mouse4mC-BGRU [39], by 0.0308 over the i4mC-Mouse [37], and by 0.0559 over the 4mCpred-EL [36]. The MultiScale-CNN-4mCPred outperformed Mouse4mC-BGRU [39] by 0.0429 MCC, the i4mC-Mouse [37] by 0.0609 MCC, and the 4mCpred-EL [36] by 0.1099 MCC. Therefore, according to the performances over both cross-validation and the independent test, the MultiScale-CNN-4mCPred is superior to these three methods. We compared MultiScale-CNN-4mCPred with the newly developed method 4mCPred-CNN [38] which is implemented into a user-friendly webserver: http://nsclbio.jbnu.ac.kr/tools/4mCPred-CNN/. We uploaded the independent set to the webserver of the 4mCPred-CNN [38] for conducting prediction. We counted the predicted results under various thresholds, as listed in Table 5. As a comparison, we also listed performance of MultiScale-CNN-4mCPred under various thresholds. The MultiScale-CNN-4mCPred was superior to the 4mCPred-CNN [38] in value of Acc and MCC. These results indicated that MultiScale-CNN-4mCPred is a competitive and advanced computational method for predicting DNA 4mC sites.

**Table 3:** The performance over the 10-fold cross-validation

	Sn	Sp	Acc	MCC
4mCpred-EL*	0.8040	0.7870	0.7950	0.5910
i4mC-Mouse*	0.6831	0.9020	0.7930	0.6510
Mouse4mC-BGRU*	0.7940	0.8400	0.8100	0.6200
MultiScale-CNN-4mCPred	0.8008	0.8294	0.8166	0.6335

*Result came from the references [36, 37, 39]

**Table 4:** The performance over the independent test

	Sn	Sp	Acc	MCC
4mCpred-EL*	0.7572	0.8251	0.7910	0.5840
i4mC-Mouse*	0.8071	0.8252	0.8161	0.6330
Mouse4mC-BGRU*	0.8000	0.8500	0.8250	0.6510
MultiScale-CNN-4mCPred	0.8563	0.8375	0.8469	0.6939

*Result came from the references [36, 37, 39]

**Table 5:** Comparison with 4mCPred-CNN over the independent test for various thresholds

Method	4mCPred-CNN				MultiScale-CNN-4mCPred			
	Sn	Sp	Acc	MCC	Sn	Sp	Acc	MCC
0.1	0.9250	0.3438	0.6344	0.3302	0.9563	0.4875	0.7219	0.5024
0.2	0.8500	0.6063	0.7281	0.4704	0.9125	0.6063	0.7594	0.5449
0.3	0.7875	0.7375	0.7625	0.5257	0.9000	0.6938	0.7969	0.6068
0.4	0.6813	0.8000	0.7406	0.4847	0.8750	0.7625	0.8188	0.6416
0.5	0.6125	0.8625	0.7375	0.4906	0.8563	0.8375	0.8469	0.6939
0.6	0.4938	0.9125	0.7031	0.4474	0.7625	0.8688	0.8156	0.6348
0.7	0.3813	0.9438	0.6625	0.3931	0.6875	0.8875	0.7875	0.5869
0.8	0.2688	0.9750	0.6219	0.3443	0.6375	0.9313	0.7844	0.5950
0.9	0.1563	0.9875	0.5719	0.2586	0.4625	0.9625	0.7125	0.4907

### Contributions of CNNs to 4mC prediction

We also investigated the contributions of CNNs with different scales to 4mC prediction. Table 6 showed the performance of removing a CNN from the MultiScale-CNN-4mCPred each time. Exclusion of a scale CNN caused the performance to decrease. For example, excluding the CNN with the size 3 made Sn decrease, excluding the CNN with the size 5 made Sp decrease, and excluding the CNN with the size 7 made both Sn and Sp decrease. Table 7 showed the performance of the MultiScale-CNN-4mCPred with the single-scale CNN. Different scale CNNs contributes differently. The CNN with the size 3 contributed mainly to Sn, the size 5 CNN to Sp, and the size 7 CNN to Sp and then to Sn. Therefore, the multi-scale CNNs contributed jointly to 4mC prediction, which is a reason to use it.

**Table 6:** The performance on excluding a scale CNN

Removing scale	Sn	Sp	Acc	MCC
3	0.8000	0.8562	0.8281	0.6572
5	0.8500	0.8187	0.8343	0.6690
7	0.8375	0.8250	0.8312	0.6625

**Table 7:** The performance at single-scale CNN

Used scale	Sn	Sp	Acc	MCC
3	0.8750	0.7688	0.8219	0.6474
5	0.7875	0.9000	0.8437	0.6918
7	0.7875	0.8750	0.8312	0.6651

### Generalized ability

We also tested the proposed method for the ability to predict 4mC sites in other species. We downloaded 6 training and 6 independent datasets respectively from six species: A. thaliana, C. elegans, D. melanogaster, E. coli, G. pickeringii, and G. subterraneus at http://thegleelab.org/Meta-4mCpred/4mCPredData.html [34]. The numbers of samples in the training and independent datasets were 3956 and 2500 in A. thaliana, 3108 and 1500 in C. elegans, 3538 and 2000 in D. melanogaster, 776 and 268 in E. coli, 1138 and 400 in G. pickeringii, and 1812 and 700 in G. subterraneus. We used the training dataset to train MultiScale-CNN-4mCPred, and used the independent dataset from the same species to test the trained model. Table 8 listed performances over independent datasets. In terms of MCC, the MultiScale-CNN-4mCPred performed better than both DeepTorrent [44] and 4mCPred [26] over three species: A. thaliana, C. elegans and D. melanogaster, performed worse than DeepTorrent [44] over three species: E. coli, G. pickeringii, and G. subterraneus. The first three species and mouse are eukaryote, while the last three species are prokaryote. The results indicated that MultiScale-CNN-4mCPred would be more suitable to predict 4mC sites eukaryote than in prokaryote.

**Table 8:** Comparison with DeepTorrent and 4mCPred over six species

Method	Species	Sn	Sp	Acc	MCC
MultiScale-CNN-4mCPred	*A. thaliana*	0.7792	**0.8440**	**0.8116**	**0.6245**
	*C. elegans*	0.8467	**0.8987**	**0.8727**	**0.7463**
	*D. melanogaster*	**0.9060**	**0.8850**	**0.8955**	**0.7912**
	*E. coli*	0.8358	**0.8731**	0.8545	0.7094
	*G. pickeringii*	0.7150	0.8000	0.7575	0.5169
	*G. subterraneus*	0.7457	0.8400	0.7929	0.5883
DeepTorrent*	*A. thaliana*	**0.8182**	0.7803	0.7992	0.5989
	*C. elegans*	**0.9038**	0.8077	0.8558	0.7149
	*D. melanogaster*	0.8898	0.8220	0.8559	0.7135
	*E. coli*	**0.9231**	0.8462	**0.8846**	**0.7715**
	*G. pickeringii*	**0.8684**	**0.9474**	**0.9079**	**0.8183**
	*G. subterraneus*	0.8333	**0.9167**	**0.8750**	**0.7526**
4mCPred*	*A. thaliana*	0.7652	0.7652	0.7652	0.5300
	*C. elegans*	0.8558	0.7885	0.8221	0.6500
	*D. melanogaster*	0.8390	0.8136	0.8263	0.6500
	*E. coli*	0.8462	0.8077	0.8269	0.6500
	*G. pickeringii*	**0.8684**	0.6842	0.7763	0.5600
	*G. subterraneus*	**0.9167**	0.7500	0.8333	0.6800

*Result came from the reference [26, 44], and the bold highlighted the best values in the corresponding species

## Conclusions

The 4mC is one of the epigenetic mechanisms. Precise and large-scale detecting 4mC sites is still challenging. We presented a multi-scale CNNs and adaptive embedding-based method called MultiScale-CNN-4mCPred for predicting 4mC sites in the mouse genomes. The MultiScale-CNN-4mCPred reached the state-of-the-art performance in the mouse genome, and showed curtain ability to predict DNA 4mC across species. This method is end-to-end without manual feature extraction, and is simple to realize. Different scales reflect different characterizations of 4mC. The MultiScale-CNN-4mCPred failed to discover the biological meaning of the different scale characterization, and textual semantic of single nucleotide residue. We also developed a user-friendly web application which is convenient to predict 4mC sites. The proposed method and the developed tool are beneficial to epigenetic research. In the future, we shall employ the Transformer [45] or the BERT [46] to improve the semantical representation of sequences, and exploit attention mechanism to promote interpretability of models.

## Methods

**Fig. 1:**
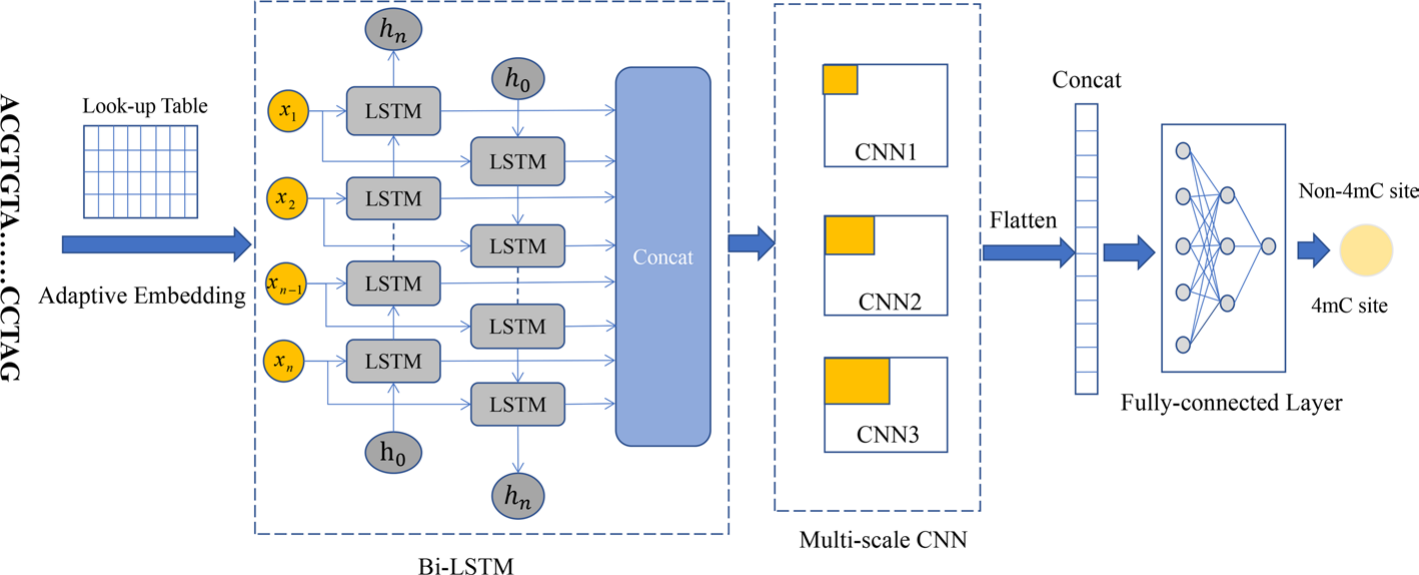
Workflow of the MultiScale-CNN-4mCPred

As shown in Fig. 1, the presented MultiScale-CNN-4mCPred is a deep neural network-based method, consisting mainly of adaptive embedding [39], bi-directional long short-term memory (Bi-LSTM) [47], multi-scale CNNs [48, 49], and three fully-connected layers. The DNA letter sequences were first transformed into sequences of integers. Then, the adaptive embedding layer was used to map sequences of integers into continuous vectors. The Bi-LSTM layer was used to extract contextual semantics from DNA sequences. The multi-scale CNN layer was used to extract local features through multiple kernels of different scales. To reduce or avoid overfitting, the dropout was attached at the end of the multi-scale CNN layer and the first fully-connected layer, respectively. The multi-scale representations were concatenated to be fed into three fully-connected layers. The final output represented probabilities of predicting inputs as 4mC. The MultiScale-CNN-4mCPred is an end-to-end learning model. That’s to say, the DNA sequence were fed into the MultiScale-CNN-4mCPred and the predictive results were directly returned without any manual operations. The parameters in each layer of the MultiScale-CNN-4mCPred were listed in Table 9. The total number of trainable parameters was 29661.

**Table 9:** Number of parameters and shape of output in the proposed

Layers	Parameters	Output
Adaptive embedding	32	[None, 41, 8]
Bi-directional LSTM	720	[None, 41,12]
Multi-Scale CNN_3	333	[None, 39, 9]
Multi-Scale CNN_5	549	[None, 37, 9]
Multi-Scale CNN_7	765	[None, 35, 9]
Dropout_3	0	[None, 39, 9]
Dropout_5	0	[None, 37, 9]
Dropout_7	0	[None, 35, 9]
Concat	0	[None,111, 9]
Flatten	0	[None, 999]
Dense(27)	27,000	[None, 27]
Dropout	0	[None, 27]
Dense(9)	252	[None, 9]
Dense(1)	10	[None, 1]

### Character encoding

The DNA sequence consisted of four characters: A, T, G, and C. The deep neural network cannot directly process the character sequences. Therefore, the character must be converted into numerical sequences. Here, we defined a map as follows:

1 $$f(c) = \left\{ {\begin{array}{*{20}l} 0 \hfill & {if\; c \;was\; A} \hfill \\ 1 \hfill & {if\;c \;was \;G} \hfill \\ 2 \hfill & {if\;c\; was\; C} \hfill \\ 3 \hfill & { if\;c \;was\; T} \hfill \\ \end{array} } \right.$$

### Adaptive embedding

Embedding is a way to transform discrete variables into continuous representations [50]. In neural networks, the embedding technique not only reduces the spatial dimension of discrete variables, but also meaningfully characterizes the variable. For example, the word2vec [42, 43] is a frequently used embedding algorithm in the field of natural language processing, where an interesting and famous example is that vector (king) − vector (man) + vector (women) ≈ vector (queen). On the contrary, the traditional one-hot encoding method translated each word into a binary vector, which has two limitations. Firstly, when the vocabulary was large, the vector generated by one-hot might be very sparse. Secondly, the one-hot encoding assumed that words were independent of each other. Thus, similarities between words could not be measured. We used adaptive embedding [39] as the first layer of the MultiScale-CNN-4mCPred.

### CNN

CNN is a well-known neural network architecture [48]. CNN first appeared in the LeNet-5 which was a multi-layer neural network [51, 52] and was used to distinguish handwritten digits. The LeNet-5 used the backpropagation algorithm for training. The CNN attracted considerable attention for the AlexNet [53], a deep CNN neural network architecture that reduced significantly the error rate of image classification in comparison with other state-of-the-art methods. Since then, many variants of deep CNN architecture have been proposed, such as ZFNet [54], VGGNet [55], GoogleNet [56], and ResNet [57]. It has widely been demonstrated that these variants are of effectiveness and efficiency in most cases. For example, the ResNet [57] won the champion of ImageNet Large Scale Visual Recognition Challenge (ILSVRC) in 2015. CNN is becoming one of the most popular and practically used components in the field of deep learning.

The CNN contained three basic components: convolution, pooling, and activations [58]. The aim of the convolution is to compute a feature map. Each neuron in the feature map is linked to a region of neighboring neurons in the previous layer which is generally called the receptive field. The kernel (also called filters) convolves with receptive fields to generate a feature map. The receptive fields slide at a certain stride in horizontal or vertical directions to generate various convolved results. The kernel is learnable and shared by all receptive fields. Various kernels yielded various feature maps. In most cases, multiple kernels are used to characterize the studied object from multiple perspectives. The convolved results were non-linearly transformed by the activation function. The commonly used activation functions include Sigmoid [59], Tanh [60], and ReLU [61], The pooling is to reduce the dimension of the feature map, and thus reduced the complexity of training deep neural networks. The pooling included max pooling, min pooling, and mean pooling.

### Bi-directional long short-term memory

Recurrent neural network (RNN) is a different architecture of neural network from the CNN. At the time step t in the RNN, the state of the hidden node ${h}^{(t)}$ was not only associated with the current input ${x}^{\left(t\right)}$, but also with the hidden state ${h}^{\left(t-1\right)}$ at the previous time step t-1, which was computed by

2 $$h^{t} = \sigma \left( {W^{hx} x^{\left( t \right)} + W^{hh} h^{{\left( {t - 1} \right)}} + b_{h} } \right)$$

where $W^{hx}$ denoted the linking weight between the input and the hidden state, and ${W}^{hh}$ was the linking weight between the hidden states. The output at the time step t was computed by

3 $${\widehat{y}}^{(t)}=soft max\left({W}^{yh}{h}^{\left(t\right)}+{b}_{y}\right)$$

where ${W}^{yh}$ denoted linking weight between output and hidden state, ${b}_{y}$ denotes bias. The marked trait of the RNN was that all the nodes shared linking weights. The RNN is especially suitable for sequence analysis. Although RNN was capable of solving the order problem of neural network in time-based sequence, the RNN still had some limitations [62, 63]. The major limitation was to vanish and to explode for the gradient. The LSTM [64] solved well this limitation of the early RNN by replacing the traditional nodes of the hidden layer with a memory cell which was a unit of computation [65].

The common long short-term memory (LSTM) cell contained input, the previous hidden state, the previous cell state, the current hidden state, the current cell state, a candidate cell state, and three gates: forget gate, input gate, and output gate. The cell state was equivalent to the path of information transmission, which was the horizontal line with only some minor linear interactions, running straight down the entire chain, so that the previous information could flow along the sequence unchanged. The cell state was viewed as the "memory" of the neural network [62]. In theory, the cell state could transmit the relevant information forever. The addition and the removal of information in the sequence were controlled by the gates. The forget gate determined what information was preserved from the cell state. The forget gate outputted a number between 0 and 1, 0 representing completely getting rid of the information and 1 completely keeping it [62]. The input gate decided which value was updated. The new candidate cell state was created by the tanh function. The cell state was updated by multiplying the previous cell state by the forget gate, and by multiplying the candidate cell state by the input gate. The hidden state was updated by multiplying the output gate by tanh of the cell state. All the computations in each LSTM cell were listed as follows:

4 $$g^{\left( t \right)} = \emptyset \left( {W^{gx} x^{\left( t \right)} + W^{gh} h^{{\left( {t - 1} \right)}} + b_{g} } \right)$$

5 $$i^{\left( t \right)} = \sigma \left( {W^{ix} x^{\left( t \right)} + W^{ih} h^{{\left( {t - 1} \right)}} + b_{i} } \right)$$

6 $$f^{\left( t \right)} = \sigma \left( {W^{fx} x^{\left( t \right)} + W^{fh} h^{{\left( {t - 1} \right)}} + b_{f} } \right)$$

7 $$o^{\left( t \right)} = \sigma \left( {W^{ox} x^{\left( t \right)} + W^{oh} h^{{\left( {t - 1} \right)}} + b_{0} } \right)$$

8 $$s^{\left( t \right)} = g^{\left( t \right)} \odot i^{\left( t \right)} + s^{{\left( {t - 1} \right)}} \odot f^{\left( t \right)}$$

9 $$h^{\left( t \right)} = \emptyset \left( {s^{\left( t \right)} } \right) \odot o^{\left( t \right)}$$

In equations above, ${W}^{fx}$ and ${W}^{fh}$ denoted the linking weight between the input and the forget gate as well as the linking weight between the forget gate and the hidden state. ${W}^{ix}$, ${W}^{ih}$, ${W}^{gx}$ and ${W}^{gh}$ denoted the linking weights between the input and the input gate, between the input gate and the hidden state, between the candidate cell and the input, as well as between the candidate cell and the hidden state, respectively. Besides, ${W}^{ox}$ and ${W}^{oh}$ denoted the linking weight between the input and the output gate, as well as between output gate and hidden state, respectively. The symbols $\sigma$ and $\varnothing$ represented the activation function sigmoid and tanh, respectively, and the symbol $\odot$ represented the point by point multiplication of vectors.

The LSTM allowed the information to flow from the past to the future. In some cases, the output was not only related to the previous input, but also might be related to the future input. A Bi-LSTM proposed by Schuster et al. [47] addressed well the issue.

### Dropout and flatten layer

The dropout was pioneered by Hinton et al. [66]. The dropout is that in the training stage, a certain proportion of the neurons were dropped out and parameters of only preserved neurons were updated. The aim of designing dropout have two-fold: avoiding overfitting and reducing the complexity of computation. Due to its effectiveness and efficiency, the dropout is increasingly becoming a frequently used trick in the deep learning area [67–69].

The flatten layer is to play the role of a bridge, which links the fully-connected layer to the non-fully connected layer. The flatten layer transformed the output of non-fully connected layer into one dimension, so that it could link to the subsequent fully-connected layer. The flatten layer has no trainable parameters. The fully-connected layer was similar to the hidden layer in the multilayer perceptron, where each neuron was connected to all the neurons in the preceding layer.

## Datasets

For a fair comparison with the state-of-the-art methods, we used the same dataset as Mouse4mC-BGRU [39], 4mCPred-CNN [38], i4mC-Mouse [37], and 4mCPred-EL [36]. The dataset originated from the MethSMRT [70], which was the first database to deposit DNA 6mA and 4mC sites. The process of MethSMRT [70] collecting the dataset was briefly described as follows. The MethSMRT [70] firstly retrieved the SMRT sequencing data from the NCBI Gene Expression Omnibus (http://www.ncbi.nlm.nih.gov/geo/) [71] and Sequence Read Archive (http://www.ncbi.nlm.nih.gov/sra) [72] and then employed PacBio SMRT analysis platform which is available at http://www.pacb.com/products-and-services/analytical-software/smrt-analysis/analysis-applications/epigenetics/ to detect 6mA and 4mC site. Reads with less than 50 nucleotides (nt), or a low-quality region (read score < 0.75 by default) were filtered out by using SFilter. The reads left were aligned to the reference genome by pbalign. The sites with less than 25-fold coverage per strand or with less than 20 modification scores were removed. The MethSMRT [70] hosted methylations of 156 species, including 7 eukaryotes and 149 prokaryotes. The DNA sequences were divided into windows of 41 base pairs with the cytosine at the center of it. The windows containing the 4mC sites were considered as positive samples and others were as negative ones. To reduce or remove the influence of sequence homology on the methods, the CD-HIT programming [73] was used to cluster the original sequences. The sequence identity was set to 0.7. Therefore, among the preserved sequences, the sequence identity between any two was less than 0.7. The same number of negative samples as the positive samples were randomly selected to a keep balance between them. All the samples were divided into the training set and the testing set. The training set consisted of 746 positive and 746 negative samples, while the testing set consisted of 160 positive and 160 negative samples.

## Evaluation metrics

We adopted Sensitivity (Sn), Specificity (Sp), Accuracy (Acc), and Matthews correlation coefficient (MCC) for evaluating the performances, which were respectively defined as follows:

10 $$Sn=\frac{TP}{TP+FN}$$

11 $$Sp=\frac{TN}{TN+FP}$$

12 $$Acc=\frac{TP+TN}{TP+TN+FP+FN}$$

13 $$MCC=\frac{TP\times TN-FP\times FN}{\sqrt{\left(TP+FP\right)\left(TP+FN\right)\left(TN+FP\right)\left(TN+FN\right)}}$$

where TP represented the number of the true 4mC samples that were correctly predicted to be 4mC, TN the number of non-4mC samples that were correctly predicted to be non-4mC, FP the number of non-4mC samples that were incorrectly predicted to be 4mC, and FN the number of 4mC samples that were incorrectly predicted to be non-4mC.

The MultiScale-CNN-4mCPred was implemented by Tensorflow 2.9.1 framework, and all scripting programs were written via Python 3.8.12. The entire project was run on a Windows 10 system configured with 2.42 GHz CPU, 2 GB GPU, and 16 GB RAM. The learnable parameters in MultiScale-CNN-4mCPred were randomly initiated. 20% of the training set is randomly sampled as the validation set.

## Web server

For the purpose of conveniently using the MultiScale-CNN-4mCPred for 4mC site prediction, we implemented the proposed method into a user-friendly web server, which is freely accessible at http://www.biolscience.cn/MultiScale-CNN-4mCPred/. The web server is very easy to use. Users paste or upload DNA sequences in the FASTA format, and then click the “submit” button. The web server will perform predictions and return the predicted results to users.

## Data Availability

The data is available at: http://www.biolscience.cn/MultiScale-CNN-4mCPred/, and the source code is available at: https://github.com/paomian97/MultiScale_CNN_4mCPred.
